# Sphingosine-1-phosphate hinders the osteogenic differentiation of dental pulp stem cells in association with AKT signaling pathways

**DOI:** 10.1038/s41368-022-00173-5

**Published:** 2022-04-22

**Authors:** Bongkun Choi, Ji-Eun Kim, Si-On Park, Eun-Young Kim, Soyoon Oh, Hyuksu Choi, Dohee Yoon, Hyo-Jin Min, Hyung-Ryong Kim, Eun-Ju Chang

**Affiliations:** 1 Department of Biomedical Sciences, Asan Medical Center, University of Ulsan College of Medicine, Seoul, 05505 Korea; 2 Stem Cell Immunomodulation Research Center, Asan Medical Center, University of Ulsan College of Medicine, Seoul, Korea; 3 Department of Pharmacology, College of Dentistry, Jeonbuk National University, Jeonju, Korea; 4 Department of Biochemistry and Molecular Biology, Asan Medical Center, University of Ulsan College of Medicine, Seoul, Korea

**Keywords:** Stem-cell differentiation, Lipid signalling

## Abstract

Sphingosine-1-phosphate (S1P) is an important lipid mediator that regulates a diverse range of intracellular cell signaling pathways that are relevant to tissue engineering and regenerative medicine. However, the precise function of S1P in dental pulp stem cells (DPSCs) and its osteogenic differentiation remains unclear. We here investigated the function of S1P/S1P receptor (S1PR)-mediated cellular signaling in the osteogenic differentiation of DPSCs and clarified the fundamental signaling pathway. Our results showed that S1P-treated DPSCs exhibited a low rate of differentiation toward the osteogenic phenotype in association with a marked reduction in osteogenesis-related gene expression and AKT activation. Of note, both S1PR1/S1PR3 and S1PR2 agonists significantly downregulated the expression of osteogenic genes and suppressed AKT activation, resulting in an attenuated osteogenic capacity of DPSCs. Most importantly, an AKT activator completely abrogated the S1P-mediated downregulation of osteoblastic markers and partially prevented S1P-mediated attenuation effects during osteogenesis. Intriguingly, the pro-inflammatory TNF-α cytokine promoted the infiltration of macrophages toward DPSCs and induced S1P production in both DPSCs and macrophages. Our findings indicate that the elevation of S1P under inflammatory conditions suppresses the osteogenic capacity of the DPSCs responsible for regenerative endodontics.

## Introduction

Numerous clinical conditions, including inflammation, congenital malformation, cancer, aging, osteoporosis, and diabetes, can cause oral bone defects that eventually lead to aggravated bone loss and disrupted bone repair.^1–4^ Bacterial invasion into the pulp evokes pulpal inflammation represented by neutrophil infiltration and the sequential infiltration of macrophages and lymphocytes.^5^ Among the immune cells, macrophages predominate in inflamed pulp tissues and produce cytokines, such as interleukin 1β (IL-1β) and tumor necrosis factor α (TNF-α), which are particularly elevated in inflamed pulp.^6,7^ This evidence implies that macrophages play a crucial role in eliciting pulpal inflammation and thereby contribute to pathologic transitions, such as oral bone defects and bone loss.

Novel bone regenerative strategies are highly anticipated to be able to overcome bone loss, which remains one of the most challenging problems in dentistry. Recently, stem cell-based regeneration, and the improved regenerative ability of these cells in vivo, has been applied to regenerative therapeutic approaches. In the area of the stem cell-based bone regeneration, dental pulp stem cells (DPSCs) have demonstrated clinical relevance including cell therapies in the odontoiatric and orthopedic areas. DPSCs are considered to be a type of mesenchymal stem cell (MSC) which shows higher proliferation rate than bone marrow-derived stem cells (BMMSCs). They also display multidifferentiation potentials, including myogenic, adipogenic, neurogenic, chondrogenic, and osteogenic/odontogenic lineages.^8,9^ The cumulative evidence to date suggests that, in addition to odontoblastic differentiation, DPSCs can also produce innovative dental materials,^10–13^ and differentiate into osteoblast precursors. In turn, these osteoblast precursors differentiate into the functional osteoblasts that generate an extracellular and mineralized matrix that can repair bone loss.^8,14^ This DPSC characteristic is the key to future technologies in bone regeneration.^15,16^ Compared with other MSCs, such as BMMSCs, DPSCs are readily and non-invasively accessible from discarded or extracted teeth. Thus, the use of DPSCs does not implicate major ethical issues.^17^ As such, DPSCs provide valuable source of easily accessible stem cells for future tissue engineering and regenerative medicine strategies.

Bioactive lipids have been known to play various roles in energy metabolism, the cardiovascular system, and aging. These bioactive lipid mediators, including acylglycerol derivatives, lysophospholipids, endocannabinoids, and sphingolipids, all have potent biological signaling functions.^18–20^ One of the novel strategies for the improvement of dental regenerative therapy with stem cells utilizes sphingosine-1-phosphate (S1P) and its associated cellular signals.^21^ This pathway regulates various cell signaling pathways, including complex inflammatory processes, proliferation, cell differentiation, migration, and survival.^21–24^ S1P is formed via the enzymatic activity of sphingosine kinase 1 (SPHK1) and 2 (SPHK2) which phosphorylate the sphingosine.^21^ and most of its biological effects are mediated via the signaling processes of five different cell membrane G-protein-coupled receptors, namely, S1PR1 to S1PR5.^25^ Previous studies have suggested that S1P plays a crucial role in bone regeneration through its enhancement of the osteogenic differentiation of osteoblast-like cells^26,27^ and that it ameliorates osseous tissue growth in vivo.^28,29^ On the other hand, an animal model study has found that FTY720, which is an S1PR1-binding molecule, activates the re-migration of osteoclast precursors from the surface of the alveolar bone to the blood vessels and reduces the osteoclastogenesis caused by periodontitis. These findings suggest that the crosstalk between S1P/S1PR1 may play a role in osteoclast formation.^30^ However, whether S1P or S1P receptor (S1PR) signaling plays a role in the DPSC-driven osteogenesis remains unknown.

In our present analyses, we explored the function of S1P/S1PR elicited signaling in DPSC-driven osteogenesis. We also investigated the potency of S1P in reducing the osteogenic capacity of DPSCs in vitro and clarified the underlying signaling mechanisms by which S1P/S1PR controls AKT signaling in the S1P-mediated attenuation of DPSC osteogenesis. In addition, we observed that the level of S1P under inflammatory conditions, particularly in relation to pro-inflammatory TNF-α cytokine stimulation, is significantly increased both in macrophages and DPSCs. Moreover, we found that TNF-α and S1P recruit macrophages to the DPSCs so that these infiltrating immune cells can induce severe inflammatory states. Taken together, our current findings suggest that S1P induced by inflammatory stimulation downregulates the differentiation potential of DPSCs toward the osteogenic phenotype.

## Results

### DPSCs treated with S1P exhibit a low differentiation rate toward the osteogenic phenotype in association with a reduction in phosphorylated AKT

**Fig. 1:**
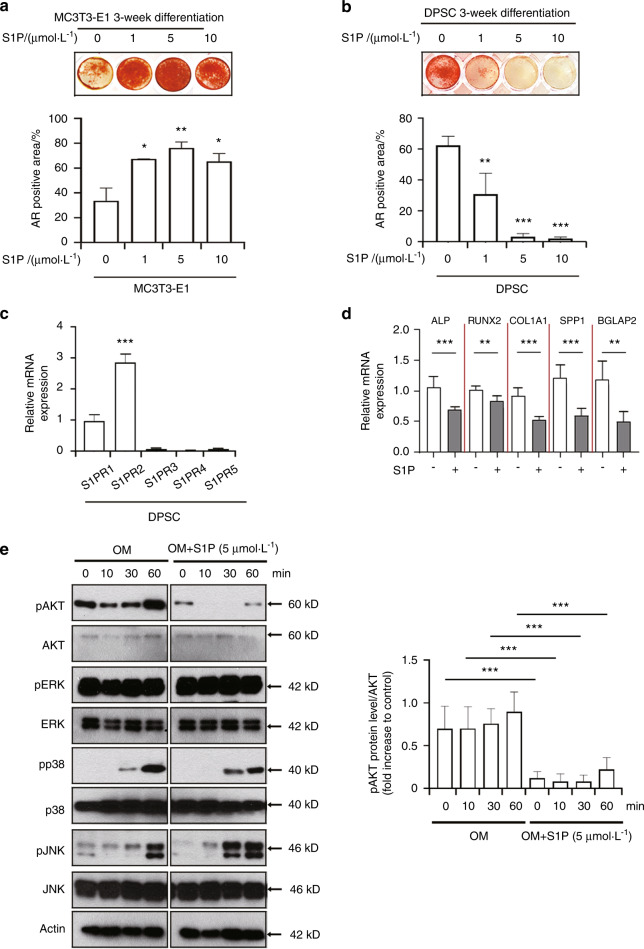
S1P hinders the osteogenesis of dental pulp stem cells (DPSCs) in association with a downregulation of osteogenic gene expression and AKT phosphorylation. **a**, **b** At 3 weeks after the induction of osteogenic differentiation in MC3T3-E1 cells (**a**) and DPSCs (**b**) with or without S1P (1, 5, or 10 μmol·L^−1^), each cell type was stained with alizarin red (AR) (upper). The graph demonstrates the AR-positive areas that were quantified from each individual culture plate (lower). **c** The transcript levels of S1PR1-5 in DPSCs at baseline were determined by qPCR analysis. **d** qPCR analysis of the mRNA expression of ALP, RUNX2, COL1A1, SPP1, and BGLAP2 in DPSCs treated with a vehicle or S1P (5 μmol·L^−1^) for 7 days. **e** DPSCs were treated with S1P (5 μmol·L^−1^) for the designated times. The protein expression of phospho-AKT (p-AKT), AKT, phospho-ERK (p-ERK), ERK, phospho-p38 (p-p38), p38, phospho-JNK (p-JNK), JNK, and β-actin was analyzed by immunoblotting (left). Densitometry quantification of p-AKT compared to AKT is represented (right); data are presented as the mean ± SEM. The experiments were at least triplicated. *P < 0.05, **P < 0.005, ***P < 0.000 5 versus the vehicle control. *P* values were obtained using the Kruskal–Wallis test (**a**–**c**), Student’s *t*-test (**d**), and one-way ANOVA (**e**)

Previous studies have indicated that S1P enhances the osteogenic conversion of osteoblast-like cells,^26,27^ but the involvement of S1P/S1P receptor signaling in the regulation of osteogenic capacity of DPSCs is still not well understood. We examined the osteoblastic differentiation potential of both MC3T3-E1 cells and DPSCs following S1P treatment by evaluating matrix mineralization, visualized by alizarin red staining (Fig. 1a, b). S1P treatment at a dose as low as 1 μmol·L^−1^ led to a significant diminishment (approximately 50%) in matrix mineralization by DPSCs compared with the control (as estimated by the alizarin red staining). This reduction was even greater when higher concentrations of S1P, with the effect being particularly evident at the 5 and 10 μmol·L^−1^ doses (Fig. 1b). In contrast, the MC3T3-E1 mouse calvaria osteoblastic cells demonstrated enhanced mineral deposition in a dose-dependent manner with S1P (Fig. 1a).

We evaluated S1P receptor expression to further examine the S1P signaling pathway as related to blocking osteogenic differentiation of DPSCs. As mentioned earlier, The major biological signals of S1P are regulated by five different S1P receptors, S1PR1–S1PR5.^25^ We evaluated the transcript levels of these receptors in DPSCs by qPCR and found that S1PR1 and S1PR2 were the most abundantly expressed. In contrast, S1PR3, 4, and 5 were undetectable (Fig. 1c). To determine whether S1P modulates the osteogenic differentiation of DPSCs, we induced this process using osteogenic media and compared the mRNA levels of the genes known to be involved in osteogenesis, including alkaline phosphatase (ALP), collagen type I alpha 1 chain (COL1A1), runt-related transcription factor 2 (RUNX2), bone gamma-carboxyglutamic acid-containing protein (BGLAP2), and secreted phosphoprotein 1 (SPP1). The transcript levels of all of these genes were significantly decreased by S1P treatment for 7 days in the presence of osteogenic media (Fig. 1d). These results revealed that S1P suppressed the osteogenic differentiation of DPSCs in association with a downregulated expression of osteogenic genes.

In general, it is well established that AKT promotes osteoblast differentiation^31^ and that the p38 MAP kinase and extracellular signal regulated kinase (ERK) pathways regulate osteogenic differentiation by modulating RUNX2.^32^ We therefore evaluated the protein expression of these genes in DPSCs following S1P treatment in the presence of osteogenic media. ERK was phosphorylated at baseline in DPSCs but the addition of S1P did not affect this (Fig. 1e). However, osteogenic media induced the phosphorylation of p38 and JNK after 60 min of exposure and this increase was not altered by S1P administration (Fig. 1e). Osteogenic media enhanced the phosphorylation level of AKT within 60 min after exposure to osteogenic media (Fig. 1e). Most importantly, the increase of activated AKT/total AKT was markedly attenuated by S1P (Fig. 1e). Collectively these results suggested that S1P reduced the osteogenic conversion capacity of DPSCs, in association with a reduction in phosphorylated AKT.

### S1PR1 and S1PR2 are involved in the S1P-mediated decrement of DPSC osteogenic capacity in association with a reduction in both activated AKT and osteoblastic markers

**Fig. 2:**
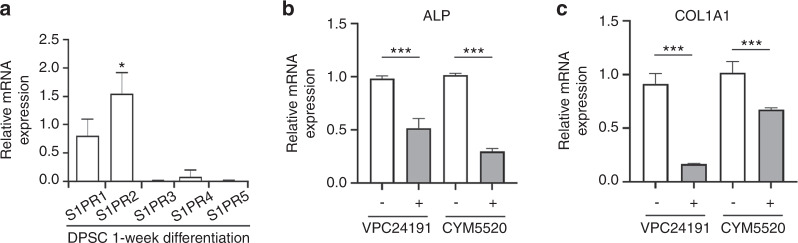
S1P agonists attenuate the osteoblastic marker expressions in DPSCs. **a** The mRNA expression levels of S1PR1–5 in DPSCs were analyzed by qPCR after treatment with osteogenic media for 7 days. **b**, **c** The expressions of ALP (**b**) and COL1A1 (**c**) in mRNA level were determined by qPCR in DPSCs treated with VPC24191 (10 μmol·L^−1^) or CYM5520 (10 μmol·L^−1^) for 7 days; Data are presented as the mean ± SEM. The experiments were at least triplicated. *P < 0.05, ***P < 0.000 5 versus vehicle control. *P* values were obtained using a Student’s *t*-test (**a**–**c**)

We next investigated the effect of osteogenic media on mRNA expression of the S1P receptor during the osteogenesis of DPSCs. We observed that osteogenic media had no obvious effect on the expression of the S1PR1 to 5 transcripts in these cells (Fig. 2a) when compared to baseline (Fig. 1c). Given the predominant expression of S1PR1 and S1PR2 in DPSCs, we determined which of these receptors was important for the S1P-mediated reduction in osteogenic differentiation in these cells when incubated in osteogenic media. We thus evaluated the mRNA expression of genes related to osteogenesis by qPCR, including ALP, and COL1A1, following exposure of the cells to VPC24191 (S1PR1/S1PR3 agonist) or CYM5520 (S1PR2 agonist) for 7 days. The activation of S1PR1/S1PR3 or S1PR2 by VPC24191 or CYM5520, respectively, significantly attenuated the transcript levels of ALP and COL1A1 (Fig. 2b, c). These results suggest that S1P may attenuates the expression of these osteoblastic markers in DPSCs via its receptor.

**Fig. 3:**
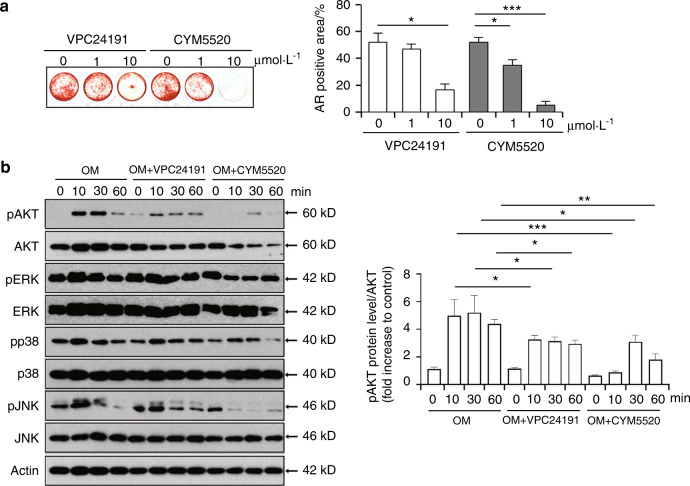
S1P agonist blocks the osteogenic differentiation of DPSCs in association with a reduction in activated AKT. **a** DPSCs were incubated with osteogenic media to stimulate their differentiation into osteogenic cells with the designated concentrations of VPC24191 or CYM5520 for 3 weeks. AR staining was then conducted (left) and the AR-positive area was quantified from each individual culture plate (right). **b** DPSCs were treated with VPC24191 (10 μmol·L^−1^) or CYM5520 (10 μmol·L^−1^) in the osteogenic media for the indicated minutes. The protein expression of p-AKT, AKT, p-ERK, ERK, p-p38, p38, p-JNK, JNK, and β-actin was analyzed by immunoblotting (left). Densitometry quantification of p-AKT compared to AKT is represented (right); Data are presented as the mean ± SEM. The experiments were at least triplicated. *P < 0.05, **P < 0.005, ***P < 0.000 5 versus vehicle control. *P* values were obtained using the Kruskal–Wallis test (**a**) and one-way ANOVA (**b**)

To gain further insights into the biological significance of the S1P receptor-mediated pathway during osteogenic differentiation of DPSCs, alizarin red staining was used to check the impact of VPC24191 and CYM5520 on the osteogenic capacity of these cells. Alizarin red staining revealed that treatment with the S1PR1/S1PR3 agonist, VPC24191, exerted a suppressive effect on matrix mineralization at a concentration of 10 μmol·L^−1^ (Fig. 3a). Similarly, CYM5520 treatment led to significant reduction in osteogenic differentiation (Fig. 3a). To elucidate the precise signaling pathway responsible for the osteogenic differentiation of DPSCs following stimulation with S1P receptor, the phosphorylation of AKT, ERK, p38, and JNK was evaluated at the protein level. Consistent with the findings for S1P demonstrated in Fig. 1d, the activation of either S1PR1/S1PR3 or S1PR2 by 10, 30, and 60 min of exposure, respectively, to VPC24191 or CYM5520 robustly suppressed the osteogenic media-induced phosphorylation of AKT (Fig. 3b). By contrast, the osteogenic media induced phosphorylation of ERK and p38 was not influenced by either VPC24191 or CYM5520, while the phosphorylation of JNK appeared to be reduced by treatment with CYM5520 (Fig. 3b). These observations suggested that S1P signaling through S1PR1/S1PR3 and S1PR2, especially through S1PR2, may reduce the osteogenic potency of DPSCs in association with the potential involvement of AKT signaling.

### AKT activation is associated with the S1P-mediated downregulation of osteogenesis-related genes and the osteogenic differentiation of DPSCs

**Fig. 4:**
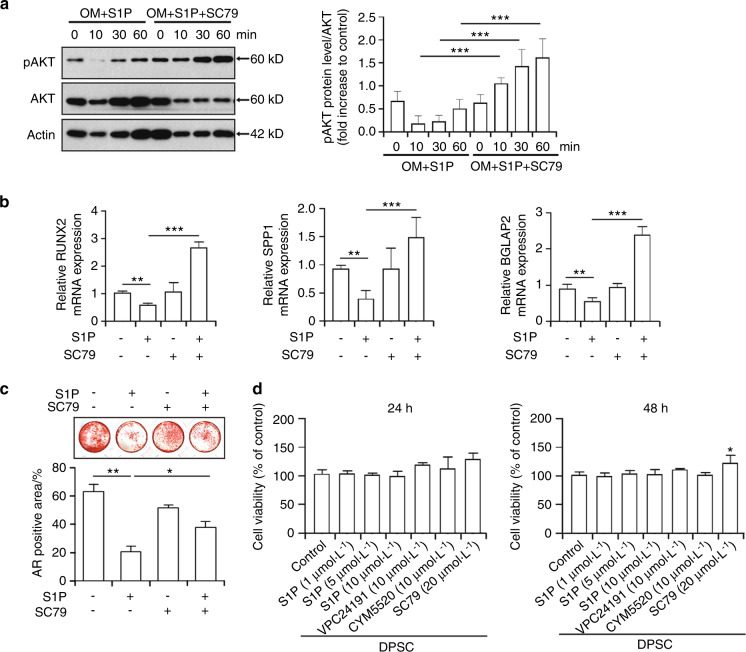
AKT activation in DPSCs is associated with the downregulation of osteogenic genes and the suppression of osteogenic differentiation mediated by S1P. **a** The protein level of p-AKT, AKT, and β-actin in DPSCs treated with SC79 (20 μmol·L^−1^) and S1P (5 μmol·L^−1^) in osteogenic media for the indicated minutes was demonstrated by a western blot (left). Densitometric quantification of p-AKT compared to AKT is represented (right). **b** The expression of RUNX2, SPP1, and BGLAP2 in mRNA level in DPSCs treated with SC79 (20 μmol·L^−1^) and S1P (5 μmol·L^−1^) for 7 days was evaluated by qPCR. **c** At 3 weeks after the induction of osteogenic differentiation in DPSCs with or without S1P (5 μmol·L^−1^) and SC79 (20 μmol·L^−1^), each cell was stained with alizarin red staining (upper). The graph demonstrates the alizarin red-positive areas, which was quantified from each individual culture plate (lower). **d** DPSCs were treated with S1P (1, 5, or 10 μmol·L^−1^), VPC24191 (10 μmol·L^−1^), CYM5520(10 μmol·L^−1^), and SC79 (20 μmol·L^−1^) for 24 or 48 h with osteogenic media. Cell viability was then measured utilizing a cell viability assay kit. Data are presented as the mean ± SEM. The experiments were at least triplicated. *P < 0.05, **P < 0.005, ***P < 0.000 5 versus the vehicle control group. *P* values were obtained using one-way ANOVA (**a**) and Kruskal–Wallis test (**b**–**d**)

Given our primary observations of an S1P-mediated inhibition of osteogenesis, accompanied by a decreased AKT activation, in DPSCs (Figs. 1–3), we next evaluated whether an AKT activator would prevent the S1P-mediated reduction in this osteogenic differentiation process. As expected, the additional exposure of DPSCs to the AKT activator SC79 augmented AKT activity within 30 min and further enhanced the phosphorylated AKT level after 60 min of treatment (Fig. 4a). In addition, the phosphorylation of AKT had been decreased after 10 and 30 min of S1P treatment and this result was consistent with the outcomes shown in Fig. 1d. SC79 alone did not show a significant effect on the mRNA level of RUNX2, SPP1, or BGLAP2 (Fig. 4b), but DPSCs treated with SC79 in the presence of S1P exhibited much higher expression of osteogenic genes compared to S1P single treatment, as determined by qPCR (Fig. 4b). Importantly, the S1P-mediated decrement in matrix mineralization was at least in part recovered by SC79 (Fig. 4c). Cell viability was not largely affected by S1P, VPC24191, or CYM5520 exposure, but SC79 modestly enhanced the cell viability of DPSCs at 48 h (Fig. 4d). In conclusion, these results indicated that the S1P-mediated attenuation of the osteogenic differentiation of DPSCs may be accompanied by AKT deactivation.

### S1P is induced by TNF-α in both DPSCs and BMMs

**Fig. 5:**
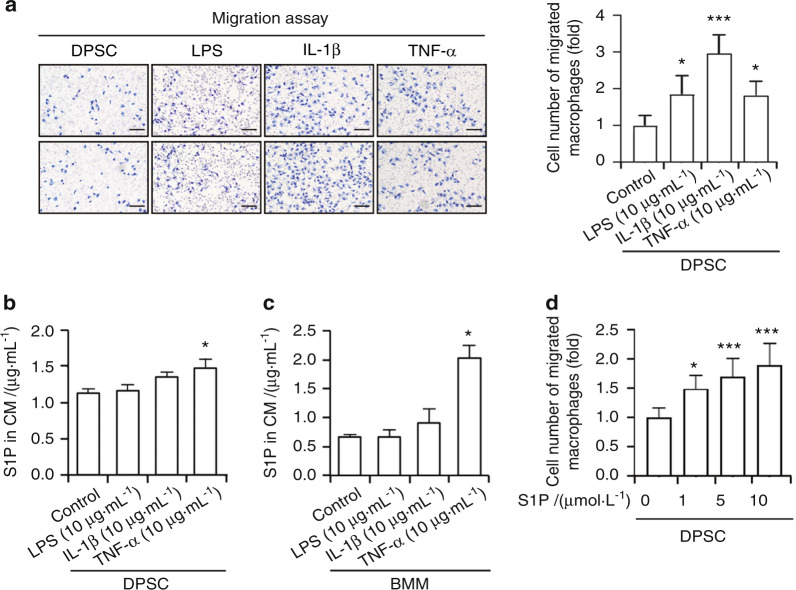
TNF-α induces S1P production in both DPSCs and bone marrow-derived macrophages. **a** Transwell assay was conducted for monitoring the migratory capacity of BMMs. DPSCs (4 × 10^4^) were placed into the lower compartment of the transwell system and treated with LPS (10 μg·mL^−1^), IL-1β (10 μg·mL^−1^), or TNF-α (10 μg·mL^−1^). BMMs (3 × 10^4^) were also loaded onto the inner membrane of the upper compartment. After 24 h of incubation, Giemsa staining was conducted to observe the migrated cells in the outer part of the upper compartment (left). Giemsa-positive cells were counted with a microscope (right). **b** DPSCs were treated with LPS (10 μg·mL^−1^), IL-1β (10 μg·mL^−1^), or TNF-α (10 μg·mL^−1^) for 24 h and the secreted S1P levels in the conditioned media (CM) were quantified by ELISA. **c** The secreted levels of S1P in the CM from BMMs were stimulated with a vehicle, LPS (10 μg·mL^−1^), IL-1β (10 μg·mL^−1^), or TNF-α (10 μg·mL^−1^) were determined by ELISA. **d** DPSCs (4 × 10^4^) were placed into the lower compartment of the transwell system and treated with the indicated concentrations of S1P. BMMs (3 × 10^4^) were also loaded onto the inner membrane of the upper compartment and, after 24 h of incubation, Giemsa staining was conducted to observe the migrated cells in the outer part of the upper compartment, and Giemsa-positive cells were counted with the microscope. Data are presented as the mean ± SEM. The experiments were at least triplicated. *P < 0.05, ***P < 0.000 5 versus the vehicle control. *P* values were obtained using the Kruskal–Wallis test (**a**–**d**)

It has been shown extensively that pro-inflammatory cytokines released during chronic inflammation such as TNF-α and IL-1β evoke bone loss,^33–35^ and that the macrophages that predominate in inflamed pulp tissues abundantly produce these cytokines and contribute to oral bone defects and bone loss.^6,7^ We therefore examined whether these pro-inflammatory mediators induce the infiltration of bone marrow-derived macrophages (BMMs) toward DPSCs and found that lipopolysaccharide (LPS) and IL-1β treatments significantly enhanced the migratory capacity of macrophages to DPSCs in a transwell assay (Fig. 5a). Likewise, TNF-α exposure also induced the migration of macrophages to DPSCs (Fig. 5a). Subsequently, we quantified S1P by enzyme-linked immunosorbent assay (ELISA) to evaluate whether these pro-inflammatory mediators induced S1P production in DPSCs. TNF-α treatment induced a higher yield of S1P production as compared with a vehicle control (Fig. 5b). By contrast, LPS had no effect on S1P production, nor did IL-1β (Fig. 5b). Similar to DPSCs, TNF-α exposure increased the production of S1P by approximately twofold in BMMs which was consistent with the prior findings of Hammad et al.,^36^ while LPS and IL-1β had no obvious effects on S1P production, as determined by ELISA (Fig. 5c). These findings suggested that TNF-α induces S1P production in both DPSCs and BMMs. To assess the involvement of S1P in macrophage chemotaxis, we performed transwell assays and found that S1P also significantly enhanced the migratory potential of macrophages toward DPSCs (Fig. 5d).

## Discussion

It has been well documented that chronic inflammation evokes periodontal disease including bone loss.^33^ For instance, pro-inflammatory immune cells and resident mesenchymal cells from an inflamed pulp site produce innumerable mediators that can activate osteoclastogenesis and/or impede osteogenesis, leading to bone resorption under inflammatory conditions.^37^ In our current analyses, we observed that the pro-inflammatory cytokine TNF-α enhances the infiltration of macrophages that elicit pulpal inflammation which eventually induced oral bone loss, and also stimulates the production of S1P in both macrophages and DPSCs (Fig. 5). In the bone marrow, TNF-α is known to stimulate receptor activator of nuclear factor kappa-Β ligand (RANKL) to directly or indirectly activate osteoclasts, leading to bone resorption.^34^ Furthermore, cytokines like IL-1β and IL-6, which are pro-inflammatory cytokines, also activated RANKL production and thereby activate osteoclasts.^35^ In prior clinical studies of periodontitis patients, non-surgical treatments were reported to lead to significant improvement in all clinical parameters related to periodontitis, followed by a decrease in the S1P level and in the activity of SPHK within 3 months.^38^ However, the linkage between oral bone loss and inflammatory mediators including S1P in dental pulp disease is not yet clear. In this context, we conclude from our current data that S1P, a mediator of complex inflammatory processes,^22^ almost completely suppresses the osteogenic potential of DPSCs, and that this is accompanied by reduction in AKT activation (Fig. 1) and in the expression of osteogenic genes, via its receptor (Fig. 2). To the best of our knowledge, our present study is the first to demonstrate the role of the S1P/S1PR pathway in the osteogenic differentiation of DPSCs.

It is notable from several lines of evidence that S1P is suggested to be involved in the osteoclastic or osteogenic differentiation in RAW264.7, SaOS-2, and MC3T3 osteoblastic cell lines, C2C12 myoblasts, and primary human femoral head osteoblastic cells^26,27,39–41^. Indeed, S1P is known to control the shifting of osteoclast precursors from the bloodstream into bone marrow cavities via its receptor pathways.^42^ Furthermore, other prior studies have reported that S1P regulates bone regeneration by enhancing the osteogenic differentiation of osteoblast-like cells^26,27,40,43^ and also that this factor promotes osseous tissue growth in vivo.^28,29^ The reason for this difference is not known but we speculate that one explanation may be a differential response to S1P between pulp stem cells and cell lines/primary human femoral head osteoblastic cells. Although S1P is known to play a crucial role in tooth regeneration, it is more likely to inhibit the osteogenic differentiation of DPSCs in an inflammatory environment and this must be taken into account in any future dental regenerative therapy strategies.

S1PR1-3 are located in most cell types, while S1PR4 and S1PR5 are limited to nerve cells and lymphocytes.^44^ One of the novel treatment strategies for dental regenerative therapy and multiple sclerosis, an autoimmune disease, has involved the targeting of S1PR1, given the reported mechanisms of S1PR1-mediated lymphocyte migration and release from lymphoid tissues.^29,45–47^ The treatment of osteoporosis through the S1PR2 regulation of osteoclasts and osteoblasts has also been tested.^48–51^ In this regard, our current analyses indicate that DPSCs express substantial amounts of both S1PR1 and S1PR2 (Fig. 1) and that VPC24191, a S1PR1/S1PR3 agonist and CYM5520, a S1PR2 agonist, significantly attenuate the expression of osteoblastic markers and inhibit the osteogenic potential of DPSCs (Figs. 2 and 3). These findings have indicated that novel drug treatment strategies targeting S1PR1/S1PR3 or S1PR2 for dental pulp disease are potential new treatment approaches. Notably in this regard however, the clinical efficacy of S1PR1/S1PR3 or S1PR2 agonists needs to be further tested in an animal model.

Akt has been demonstrated to be a downstream molecule regulated by the S1P receptor dependent signal pathways for cell survival^52^ and the S1PR-mediated PI3K/AKT signal transduction pathway has been reported to at least partially regulate S1P-induced osteoblast differentiation.^26^ Nonetheless, we here observed that the administration of S1P prohibits the activation of AKT-related signals in DPSCs under osteogenic differentiation conditions (Fig. 1) while the AKT activator SC79 prevents the S1P mediated reduction of these genes and attenuates the osteogenic differentiation of DPSCs (Fig. 4). This raises the possibility that the use of AKT activators may be a novel approach to establishing a regenerative microenvironment by maximizing the osteogenesis-related regenerative capabilities of DPSCs. Further investigations are required to evaluate the therapeutic efficacy of AKT activators, at least in an animal model. Although the influence of an AKT activator on the expression of osteoblastic markers remain unclear, our findings on the S1P-mediated AKT signaling pathway may contribute to the current understanding of the osteogenic differentiation of DPSCs.

**Fig. 6:**
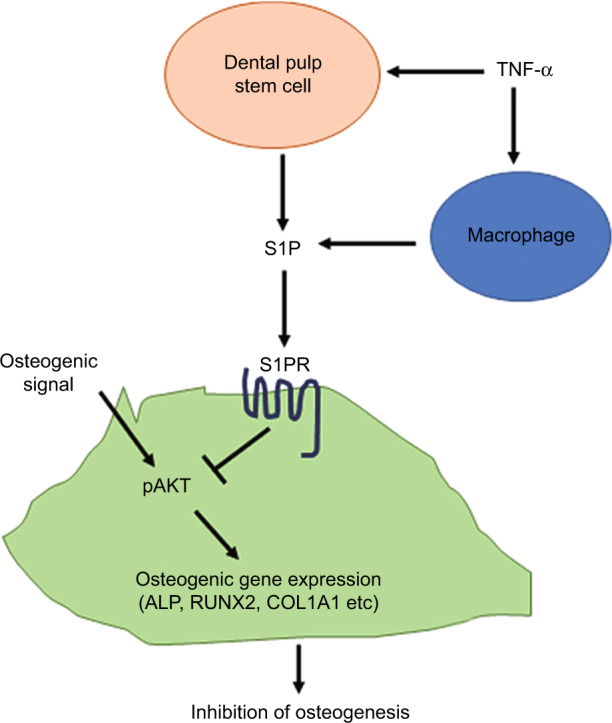
Diagram of the suggested mechanism by which S1P controls the osteogenesis of DPSC under inflammatory conditions. TNF-α induces the production of S1P in both DPSCs and macrophages. The S1P/S1P receptor signaling pathway in DPSCs reduces AKT activity and downregulates the genes related to osteogenesis, leading to an inhibition of osteogenic differentiation

In conclusion, TNF-α promotes macrophage chemotaxis and enhances S1P production. This elevated S1P level recruits more macrophages toward inflamed DPSCs and also dramatically suppresses the activation of AKT and almost completely blocks the osteogenic differentiation of these cells via S1PR1/S1PR3 or S1PR2. AKT activation may be associated with preventing the S1P-mediated downregulation of osteogenesis-related genes and blocks the attenuation of osteogenic potential. Based on our present findings, we propose a model of S1P/S1P receptor signaling pathway function in the osteogenic differentiation of DPSCs that is summarized in Fig. 6. Taken together, our current findings provide crucial new insights into S1P as a potential therapeutic target for promoting oral bone regeneration.

## Materials and methods

### Reagents

LPS (L3024; Sigma-Aldrich, St. Louis, MO), TNF-α (210-TA-020, R&D, Minneapolis, MN), and IL-1β (201-LB-005, R&D) were obtained commercially. S1P was purchased from Tocris (1370; Minneapolis, MN). VPC24191, a S1PR1/S1PR3 agonist and CYM5520, a selective agonist of S1PR2, were obtained from Sigma-Aldrich. The AKT activator SC79 was purchased from Tocris.

### Cell culture and osteogenic differentiation

The DPSCs (Lonza, PT-5025) in this study were maintained in DMEM containing 1% penicillin-streptomycin and 10% fetal bovine serum (FBS). To induce osteogenesis from DPSCs, the cells were seeded with 2 × 10^4^ cells per well density in 48-well culture plates and grown to confluence. The culture media were then replaced with osteogenic media (DMEM supplemented with 10% FBS, 0.25 mmol·L^−1^ L-ascorbic acid, 10 mmol·L^−1^ β-glycerophosphate, and 10 nmol·L^−1^ dexamethasone) and the cells were further cultured for 3 weeks, with fresh replacement of the media every 3 days. This osteogenic differentiation of DPSCs was conducted in the presence of S1P, VPC24191, CYM5520, or SC79. Bone marrow-derived macrophages (BMMs) were prepared as described previously^53^ and then cultured in alpha-minimum essential medium (α-MEM).

### Alizarin red staining

The calcific deposition in DPSCs was evaluated by alizarin red staining (Sigma-Aldrich, A5533). After 3 weeks of DPSC differentiation with osteogenic media, as described above, the cells were fixed with 4% paraformaldehyde for 30 min, and stained with 2% alizarin red solution for 30 min. ImageJ was used to measure the relative alizarin red-positive area.

### Western blotting

For western blotting analysis, cell lysates were prepared from DPSCs and were resolved by sodium dodecyl sulfate–polyacrylamide gel electrophoresis (SDS-PAGE). The lysates were then electrophoretically transferred to a polyvinylidene difluoride membrane (Bio-Rad) for immunoblotting. Non-specific interactions were blocked utilizing 5% bovine serum albumin for 1 h. The membranes were then incubated with primary antibodies against p-AKT, AKT, p-ERK, ERK, p-p38, p38, p-JNK, JNK (Cell Signaling Technology, Danvers, MA) and β-actin (Sigma-Aldrich) overnight at 4 °C, followed by incubation with the suitable secondary antibodies which were conjugated with HRP. SuperSignal West Femto Maximum Sensitivity Substrate (cat no 34095; Thermo Fisher Scientific, Waltham, MA) was used to detect the immunoreactivity. The intensity of protein band was quantified using the ImageJ Gel Analysis program.

### S1P ELISA

The concentrations of S1P in the conditioned BMM or DPSC media were measured using a commercially available S1P ELISA (cat no. MBS2700637; MyBioSource, San Diego, CA) following the manufacturer’s instructions. All samples were examined in triplicate for each experiment.

### RNA isolation and real-time PCR

QIAzol lysis reagent (Qiagen, Crawley, UK) was used to extract RNA from BMMs or DPSCs as per the manufacturer’s protocols. The RevertAid First Strand cDNA Synthesis Kit (cat no. K1622; Thermo Fisher Scientific) was used to synthesize first-strand cDNA. qPCR analysis was performed using LightCycler 480 SYBR Green I Master (04707516001; Roche, Meylan, France) with a LightCycler 480 Instrument II (Roche, Meylan, France). Glyceraldehyde 3-phosphate dehydrogenase (GAPDH) was used to normalize the expression of each gene. The results were presented as the ratio of target gene PCR product to the GAPDH level. The relative expression of each target gene was calculated via the comparative threshold cycles (2^−ΔΔCt^) method.

### Transwell assay

The migratory ability of the BMMs was observed with the transwell cell migration system (Costar, Corning, NY). Briefly, DPSCs (4 × 10^4^) were put into the lower chamber of the transwell and treated with LPS (10 μg·mL^−1^), IL-1β (10 μg·mL^−1^), TNF-α (10 μg·mL^−1^), or S1P (5 μmol·L^−1^). BMMs (3 × 10^4^) were then placed into a 5-μm pore size transwell in the upper chamber. After 24 h of incubation, the BMMs that had not migrated were removed with a cotton swab and the migrated BMMs which were attached to the outer membrane of the upper chamber were stained with Giemsa (Sigma-Aldrich). BMMs that were Giemsa positive were counted under a microscope.

### Cell viability assay

The DPSCs were placed in 96-well culture plates. When the cells reached confluence, osteogenic media was applied in the presence of S1P, VPC24191, CYM5520, or SC79 for 24 or 48 h. Quanti-MAX WST-8 Cell viability Assay Kit reagent (Biomax, Seoul, Republic of Korea) was added to media and incubated at 37 °C for 1 h. The absorbance was then measured with a VICTOR Nivo Multimode Plate Reader (PerkinElmer, Waltham, MA) at 450 nm.

### Statistical analysis

All quantitative experiments were triplicated and all results were expressed as a mean value ± SEM. A two-tailed Student’s *t*-test, one-way ANOVA, or a non-parametric Kruskal–Wallis or Mann–Whitney test was performed to assess statistical significance. A Tukey correction was conducted to adjust for multiple to one comparison. *P* values <0.05 were considered statistically significant.

## Data Availability

All of the data generated or analyzed in this study are included in this published article.
